# First empirical evidence of naturally occurring androgenesis in vertebrates

**DOI:** 10.1098/rsos.170200

**Published:** 2017-05-24

**Authors:** Miguel Morgado-Santos, Sara Carona, Luís Vicente, Maria João Collares-Pereira

**Affiliations:** 1 Centro de Ecologia, Evolução e Alterações Ambientais (cE3c), Universidade de Lisboa, 1749-016 Lisboa, Portugal; 2 Centro de Filosofia das Ciências da Universidade de Lisboa (CFCUL), Faculdade de Ciências, Universidade de Lisboa, 1749-016 Lisboa, Portugal

**Keywords:** clonal spermatogenesis, paternity analysis, microsatellite genotyping, allopolyploid complex, Cyprinidae, *Squalius alburnoides*

## Abstract

Androgenesis among vertebrates is considered a rare phenomenon, with some cases reported so far, but linked to experiments involving gamete manipulation (artificial androgenesis). Herein, we report the first empirical evidence of the natural occurrence of spontaneous androgenesis in a vertebrate, the *Squalius alburnoides* allopolyploid complex. A genetically screened random sample of a natural population was allowed to reproduce in an isolated pond without any human interference, and the viable offspring obtained was later analysed for paternity. Both nuclear and mitochondrial markers showed that the only allodiploid fish found among all the allotriploid offspring was androgenetically produced by an allodiploid male. This specimen had no female nuclear genomic input, and the sequence of the mitochondrial fragment examined differed from that of the male progenitor, matching one of the parental females available in the pond, probably the mother. The possible role of androgenesis in the reproductive dynamics of this highly successful vertebrate complex is discussed.

## 1. Introduction

Androgenesis is a reproductive mode in which the offspring produced lack maternal nuclear genomic contribution, i.e. all the genetic content of the progeny is inherited from the father (reviewed in [1–3]). It is considered a quasi-sexual form of reproduction [1], since, conversely to parthenogenesis and similarly to gynogenesis [4], it requires fertilization and syngamy, in which the oocytes serve solely as involucres to host the genetic nuclear content of the spermatozoa, via multiple mechanisms [1], being, thus, considered a form of sexual parasitism [5].

Summarizing the literature on the subject, androgenesis may be divided into two types, according to its form of occurrence: artificial androgenesis versus natural androgenesis (reviewed in [1]). Artificial androgenesis occurs when gametes are manipulated in the laboratory (a procedure used in animals with external fertilization as fish and molluscs) to produce viable androgenetic offspring, for instance by fragmenting the pronucleus of oocytes (female genome) prior to fertilization or blocking the first mitotic division of the egg. Natural androgenesis occurs in natural contexts, without any kind of manipulative intervention on animals' reproduction. Individuals derived from natural androgenesis, with a ‘paternal monopolization of parenthood’ [6], may become clones of their father after the extrusion of the maternal nuclear genome post-fertilization, typically through the polar bodies, though they normally retain the cytoplasm, mitochondria and other organelles from the oocyte [2]. In turn, two sub-types of natural androgenesis can be considered: (i) obligate androgenesis, which is an integrant part of the reproductive dynamics of certain organisms, being the main reproductive strategy of some natural populations only producing androgenetic offspring; and (ii) spontaneous androgenesis, which occurs when parents from species that reproduce sexually unexpectedly yield a certain proportion, typically low, of descendants only inheriting the paternal nuclear genome among their mainly sexually derived offspring (reviewed in [1,2]).

Natural androgenesis is considered to be a rare phenomenon, which may or may not be related to its actual incidence in wild organisms. On the one hand, with the exception of haplodiploid systems, androgenetic offspring is considered unviable in the vast majority of cases, namely due to the abnormalities associated with the ‘haploid syndrome’, being, thus, necessary that the zygote comprises more than one set of parental (in this case, paternal) chromosomes in order to be successful. This can be accomplished through paternal genome duplication (e.g. diploidization by cell fusion during the first egg division or by polyspermic fertilization) or through the production of non-haploid spermatozoa (unreduced gametes), as typically occurs in fertile hybrids or in polyploid organisms (see [1]). Indeed, most known cases of natural androgenesis involve hybridization and/or polyploidy [1,2,6]. However, on the other hand, the rarity of reports on natural androgenesis may be related to the difficulty in identifying androgens in natural populations, namely in hybrid complexes and in those cases arising from spontaneous androgenesis, since detection procedures require in-depth parentage analyses. Specifically, it is necessary to confirm a totally unique sperm-derived inheritance in the progeny, using genetic and/or cytological genomic markers for both maternal and paternal gene pools. Similarly to other quasi-sexual reproductive modes [7,8], the actual evolutionary impact of spontaneous androgenesis in wild populations has been overlooked, due to the higher extinction risk of male-cloning systems, and is poorly understood also due to the lack of data [1,2,5,9].

In animals, only a few cases of natural androgenesis have been reported (in arthropods and molluscs) [1,3], and no cases are known among vertebrates. The *Hypseleotris* carp gudgeons, recently included in a review about natural androgenesis [1], are actually a case of hybridogenesis, as clearly stated by the authors. Only two cases of spontaneous androgenesis in vertebrates have been described so far, also in fishes [10,11], but, since they involved artificial strains and/or the use of fertilization techniques, they do not represent true cases of natural androgenesis, which by definition occurs in natural contexts and in wild populations. These two cases were excluded from that recent review on natural androgenesis [1], since they are more correctly assigned to artificial androgenesis. The first case of naturally occurring spontaneous androgenesis *sensu stricto* in vertebrates is here presented and documented; it was recently found in the allopolyploid fish complex *Squalius alburnoides* in the frame of a specific study aiming to compare the reproductive success of distinct genomotypes [12].

This hybrid complex had its origin in intergeneric crosses between *Squalius pyrenaicus* females (P genome) and males from an extinct species belonging to the *Anaecypris hispanica* lineage (A genome). The hybridization event produced fertile PA hybrids, which, through crosses among themselves and backcrosses, led to the arising of an allopolyploid complex, composed by diploid (2n = 50), triploid (3n = 75) and tetraploid (4n = 100) males and females with distinct proportions of the parental genomes (=genomotypes) (reviewed in [13]). In the breeding system of this fish complex, though natural populations are highly female-biased, there is a clear sperm-dependency (*sensu* [14]), with hybrid individuals reproducing either sexually or nonsexually (*sensu* [13]). All known genomotypes are fertile, exhibiting a wide range of reproductive modes, including regular (sexual) meiosis, meiotic hybridogenesis and clonal gametogenesis (reviewed in [13]). Some allotriploid females can produce both reduced and unreduced gametes simultaneously [15], and males and females of the same genomotype may have distinct reproductive modes [16,17]. Moreover, sex ratios vary among genomotypes (e.g. allotriploids are mostly females) and geographical areas (allodiploids are mostly all male in northern populations, but all female in southern populations) (see [13]).

**Figure 1.**
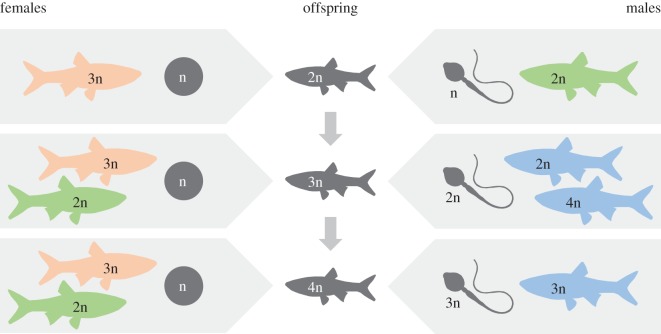
Ploidy level cascade of the reproductive framework of the *S. alburnoides* allopolyploid complex in the studied stretch of the Ocreza River (Tagus drainage, Central Portugal), illustrating the typical reproductive dynamics of northern populations dominated by triploid females. Hybrid males and females are represented in blue and pink, respectively, and males and females of the bisexual sympatric *Squalius* species are represented in green. Reproductive modes include (i) regular meiosis in *S. pyrenaicus* males and females (producing haploid P gametes) and in balanced tetraploids (producing diploid PA gametes); (ii) meiotic hybridogenesis in triploid females (producing haploid A oocytes); and (iii) clonal spermatogenesis in diploid and triploid males (producing diploid PA and triploid PAA or PPA spermatozoa, respectively). Diploid nuclear non-hybrid males (AA) are absent in the studied population (as in all northern populations) and were, thus, not included in the diagram. Both oocytes and sperm are represented in grey. 5n (and higher) offspring are unviable. The diagram illustrates well the dependence of the hybrid complex on the sympatric bisexual *Squalius* species, through the production of allodiploids, essential to the progression of the ploidy level cascade. Note that, since allotetraploids also produce diploid gametes, they could eventually replace allodiploids in the ploidy level cascade, but they are extremely rare in the vast majority of populations (see [13]).

The variety of reproductive strategies leads to changes in ploidy level through an intricate reproductive dynamics between genomotypes and illustrates well the occurrence of sexual parasitism (reviewed in [5,13]). Most genomotypes are reproductively interdependent, meaning their persistence in natural populations depends on crosses involving other genomotypes (figure 1). Moreover, the production of allodiploids is entirely dependent on crosses with bisexual species of the *Squalius* genus. Since allodiploids are crucial for the persistence of natural populations, being indispensable for the continuity of the ploidy level cascade (figure 1) and responsible for the production of allotriploids (the most common genomotype in natural populations), the persistence of *S. alburnoides* complex as a unit is, in turn, entirely dependent on the sympatric bisexual *Squalius* species.

As explained above, the production of unreduced gametes by hybrid organisms increases the likelihood of the occurrence of quasi-sexual successful reproduction, such as gynogenesis and androgenesis, which turns *S. alburnoides* complex into an excellent model to look for the existence of these unorthodox reproductive modes in the context of wild populations. Herein, using the same dataset previously published [12], which was obtained from an empirical study of a random sample from a natural population that was transferred to a pond and allowed to reproduce without any human interference, a new reproductive mode for *S. alburnoides* complex was discovered—spontaneous androgenesis—representing the first documented report of its natural occurrence among vertebrates.

## 2. Material and methods

### 2.1. Fish sampling and laboratory procedures

A random sample of *S. alburnoides* (*N* = 33) and *S. pyrenaicus* (*N* = 19) was captured in Ocreza River (Tagus drainage, Central Portugal) with short pulse and moderate voltage electrofishing (300 V, 2–4 A), during the reproductive season (April 2010), when mature individuals could be easily sexed by applying a mild pressure on the abdomen and observing the discharge of gametes. Because most *S. alburnoides* genomotypes are morphologically similar, the ploidy and genome combination of each individual were assessed in the laboratory. Individuals were anaesthetized (0.1 g l^−1^ MS-222, 0.2 g l^−1^ NaHCO_3_) and photographed on the left and right sides to be individually recognized when needed [18]. Small clips of the caudal fin were collected for genomotype assessment through flow cytometry [19] and Sanger sequencing of the β-actin gene (PCR conditions: 35 cycles of 94°C, 30 s; 55°C, 40 s; 72°C, 90 s) [20]. DNA extraction followed an adapted phenol-chloroform protocol [21].

All sampled fishes (*S. alburnoides* hybrids and *S. pyrenaicus,* PP; *N* = 52), composed of PAA (*N* = 23) and PP (*N* = 9) females, and by PA (*N* = 6), PAA (*N* = 1), PPA (*N* = 2), PPAA (*N* = 1) and PP (*N* = 10) males, were translocated to an exterior pond, under natural light and temperature conditions, in January 2011. This pond had a volume of 4200 l [300 cm length × 200 cm width × 50 cm mean depth (25–90 cm)] and was enhanced with macrophytes and with a bottom cover of small and large cobbles (2–15 cm), to provide adequate habitat conditions for the fish [22]. Two pumps and a UV lamp were used to prevent water stagnation and quality deterioration throughout the study period. Overall, habitat conditions in the pond were close to those found in Iberian rivers during seasonal drought, when fish concentrate in isolated pools [23]. Fish were fed twice a day with commercial flakes during the first month to prevent eventual lows in prey availability and facilitate adaptation to the pond conditions. The pond was monitored weekly for water pH (7–10) and inspected for dead fish (never detected) and larvae (first spotted in April). In October, parental fish and offspring were captured using electrofishing and transported to the laboratory in aerated vats. The pond was emptied to assure complete fish collection.

In the laboratory, a sample of 100 youngs-of-the-year (YOYs) was randomly selected for sex determination and paternity assessment, sacrificed with an overdose of anaesthetics (MS-222) and dissected for gonad examination, as described in [24]. Paternity was assessed through microsatellite genotyping, using nine microsatellites with high variability among cyprinids [25–27]. An extra microsatellite was haphazardly found after sequencing a genomic fragment containing the intron region of the *aminomethyltransferase* gene (AMT) (MM Coelho *et al*. 2013, unpublished data), from which the primers were designed [12]. Excepting LCO1, LCO3 and LCO4, all microsatellites were genotyped using primers with an M13 tail, as described in [28]. Complete information on the ten microsatellites used is shown in electronic supplementary material, table S1. Moreover, a mitochondrial fragment of the D-loop/control region [29] was amplified (PCR conditions: 35 cycles of 94°C, 30 s; 50°C, 30 s; 72°C, 90 s) and sequenced. Sequences were analysed in software MEGA6 [30].

## 3. Results and discussion

All 261 YOYs obtained in the pond were morphologically identified as *S. alburnoides*, with no *S. pyrenaicus* (PP) found. Flow cytometry revealed that only one of the YOYs randomly sampled (*N* = 100) was diploid, with all the others being triploid (for more results and details, see [12]), and the sequence of the *β-actin* gene revealed that the diploid individual, with 5.1 cm of standard length, was an allodiploid (PA genomotype). Further flow cytometry analyses of all the remaining YOYs (*N* = 161) revealed only triploid individuals. According to present knowledge, the only way to obtain allodiploid offspring in populations where AA males are absent (i.e. all northern populations) is through crosses between allotriploid females (which generally produce haploid A oocytes by meiotic hybridogenesis) and males of the sympatric *Squalius* species (which produce haploid sperm by regular meiosis) (figure 1), emphasizing the reproductive dependence of the hybrid complex towards the sympatric bisexual species of the *Squalius* genus. However, paternity assessment using microsatellites revealed that the nuclear P genome present in the only PA YOY found was not inherited from a PP individual (table 1). Instead, its nuclear PA genome was an exact copy of one of the *S. alburnoides* allodiploid male progenitors (PA genomotype) present in the original random sample of the natural population transferred to the pond, with all alleles from all microsatellites being a match (named PA6 in table 1). Indeed, the allodiploid YOY was male, which is consistent with an androgenetic origin.

**Table 1.** Allele comparison between the PA YOY and all PP (*N* = 10), PA (*N* = 6) and PPAA (*N* = 1) parental males present in the pond. Highlighted alleles in the list correspond to PA YOY alleles, and matching alleles with each possible parental male are shown in green. Match percentage represent the proportion of microsatellites sharing alleles between the PA YOY and each possible parental male. Male reproductive modes were taken into account when calculating match percentages: (i) PP males produce haploid sperm (P genome), meaning they would only pass half of their genome (one allele per microsatellite) to the descendant; (ii) PA males produce unreduced clonal diploid sperm (PA genome), meaning they would pass their entire genome (two alleles per microsatellite) to the descendant; and (iii) PPAA males produce reduced diploid sperm (PA genome), meaning they would pass half of their genome (two alleles per microsatellite) to the descendant.

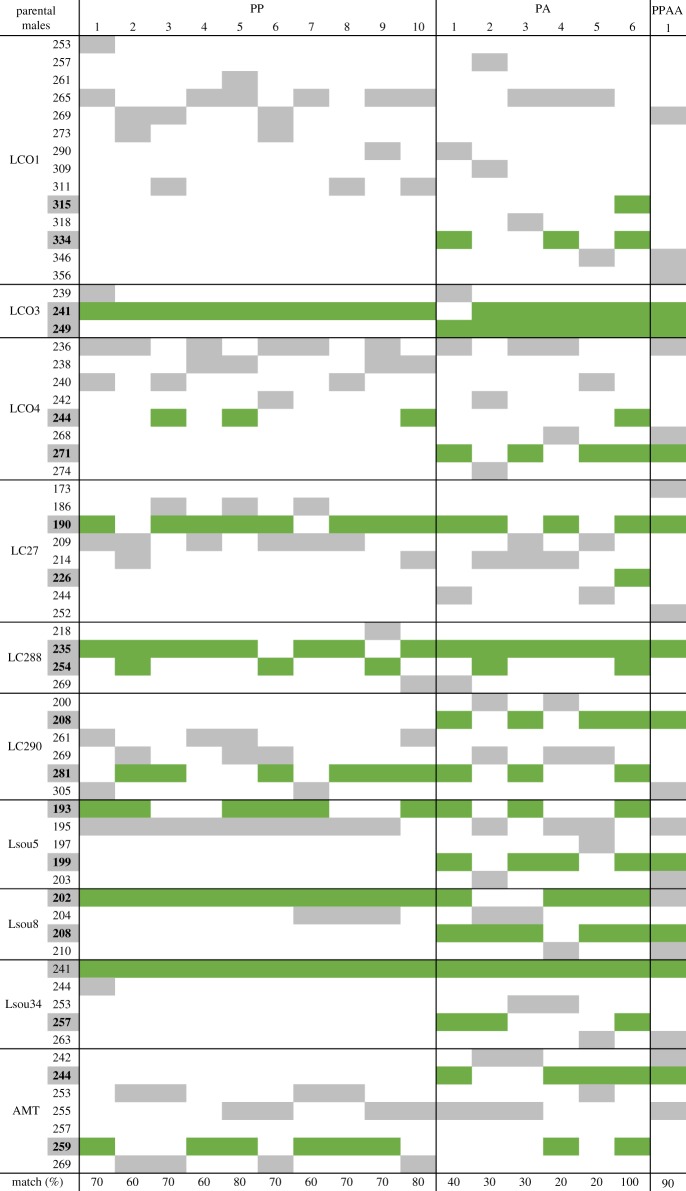

**Figure 2.**
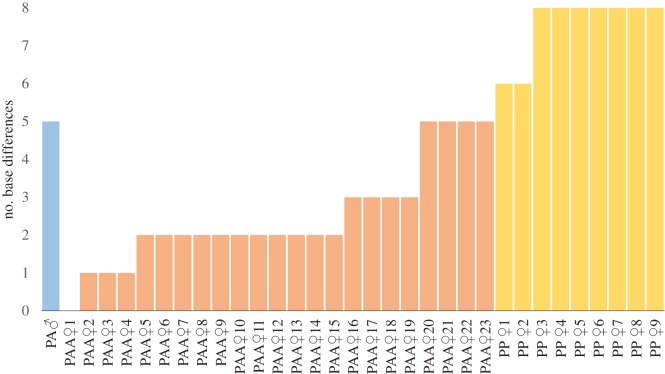
The number of base differences between the mitochondrial sequence of the androgenetic PA YOY and the mitochondrial sequences of all possible parental PAA (PAA♀1–PAA♀23; pink bars) and PP (PP♀1–PP♀9; yellow bars) females and of the PA male progenitor (PA♂; blue bar). Females were ordered according to the number of base differences towards the androgenetic PA YOY (see text for further details).

However, the referred allodiploid YOY did not share the mitochondrial DNA with his father. All SNPs present in the sequenced fragment matched one of the parental allotriploid females in the pond, probably its mother (named PAA♀1 in figure 2). It is important to note that this pair of parental fish (PAA female × PA male) produced more offspring that followed the expected reproductive modes (figure 1). They were all allotriploids (PAA), resulting from haploid oocytes (A) fertilized by unreduced spermatozoa (PA) [12]. As a side outcome from mitochondrial DNA analysis, haplotypes from *S. alburnoides* and *S. pyrenaicus* showed marked differences (figure 2), suggesting that, currently, PP females tend not to cross with hybrid males in the studied population, thus hampering mitochondrial gene flow into the hybrid complex.

To our knowledge, this is the first report of naturally occurring spontaneous androgenesis among vertebrates. Similarly to this case, both descriptions of androgenesis in vertebrates, though occurring in artificial contexts, concerned teleost fishes [10,11]. They involved hybridization and/or genome polyploidization, which facilitate the (artificial) development of androgenesis, and the observed frequency of androgenetic offspring was very low (1% and 5% [10,11]). Regarding our data, the single androgenetic individual found represented 1.0% of the total offspring genetically screened, 1.3% of the offspring of its male progenitor, 4.2% of the offspring of its female progenitor and 4.3% of the offspring of its male and female progenitors' pair (for data on other crosses, see [12]).

Although *S. alburnoides*, similarly to other hybrid complexes, undergo significant population variations regarding sex ratios, ploidy and genomotype composition, the vast majority of natural populations share their dependence on the bisexual *Squalius* species to persist. Hybrids sexually parasitize these sympatric species to produce allodiploids, being, thus, able to proceed with the ploidy level cascade to form allotriploids (figure 1) [13], the genomotype dominating most natural populations. However, recent data showed that the occurrence of natural crosses between *S. alburnoides* and *S. pyrenaicus* individuals does not seem as likely as expected, either in free-access or directional crosses [12], despite offspring production being viable in forced (totally artificial) experimental crosses (e.g. [16]). Herein, our findings report, for the first time, a route to produce allodiploid males without the involvement of the sympatric bisexual *Squalius* species, i.e. via androgenesis. Though rare, this alternative reproductive mode may guarantee the production of a sufficient proportion of allodiploid males to assure the persistence of northern populations (where most allodiploids are males; see [13]), since even a low frequency of allodiploid males seems enough to stabilize genomotype composition at an equilibrium [31]. Moreover, the androgenetic male was produced by a particular allodiploid male showing an astonishingly high reproductive success (fathering 77% of the total offspring analysed; see [12] for further details), meaning this ‘super-male’ produced a copy of himself. Being a clone, the androgen probably shared the same reproductive traits leading to the high fitness of his father, meaning that spontaneous androgenesis, even occurring at a low frequency in natural populations, may lead to the emergence of extremely successful lineages of males. This finding highlights the relevance that single individuals may have to the overall dynamics of an entire population, and challenges the view that spontaneous androgenesis, due to its low incidence, is probably insignificant to the whole reproductive dynamics of natural populations.

Regarding *S. alburnoides* hybrid complex, our findings raise the question of how frequent is this quasi-sexual reproductive mode in northern populations, which depend mainly on allodiploid males to persist. On the other hand, in most southern populations, where all allodiploids found so far are females (see [13]), the putative incidence of gynogenesis (rarely observed in artificial crosses [13]) should also be investigated, since this equivalent quasi-sexual reproductive mode for females would also make these populations become independent from the sympatric bisexual *Squalius* species. Through a combination of sexual and quasi-sexual reproductive modes, *S. alburnoides* complex would become an autonomous evolutionary unit, independent from any parental species, being able to still keep its hybrid profile and to maintain a high genetic variability.

In hybrid complexes, a remarkable diversity of reproductive strategies that overcome meiotic constraints may well be the rule and not the exception, and, thus, all such truly ‘open-systems’ pose as excellent models to study unusual reproductive systems [6,13,32]. Whenever organisms are known to produce unreduced gametes in natural populations [33], an opportunity for the emergence of quasi-sexual reproduction is settled, since the offspring may directly get the minimum double genome (diploid condition) required in the absence of the genome of the other parent (gamete). Therefore, such organisms represent valuable windows-of-opportunity to reassess the actual expression of quasi-sexual reproduction, such as spontaneous androgenesis [1,3] in natural populations, especially in taxa in which a high incidence of natural hybridization is well known and typified.

## Supplementary Material

Microsatellite details

## Data accessibility

GenBank accession numbers of DNA sequences: (i) β-actin P haplotype from Ocreza River, Tagus drainage, EF459400 [35]; (ii) β-actin A haplotype from Ocreza River, Tagus drainage, EF459401 [35]; (iii) mitochondrial D-loop of androgenetic PA YOY, KX910704; (iv) mitochondrial D-loop of androgenetic PA YOY father, KX910705; (v) mitochondrial D-loop of androgenetic PA YOY possible PAA mothers, KX910706-KX910728; and (vi) mitochondrial D-loop of androgenetic PA YOY possible PP mothers, KX910729-KX910737.

## References

[1] Hedtke SM, Hillis DM 2011 The potential role of androgenesis in cytoplasmic-nuclear phylogenetic discordance. Syst. Biol. 60, U87–U137. (doi:10.1093/sysbio/syq070) 10.1093/sysbio/syq070 21060067

[2] Pigneur LM, Hedtke SM, Etoundi E, van Doninck K 2012 Androgenesis: a review through the study of the selfish shellfish *Corbicula* spp. Heredity 108, 581–591. (doi:10.1038/hdy.2012.3) 22473310 10.1038/hdy.2012.3 PMC3356815

[3] Schwander T, Oldroyd BP 2016 Androgenesis: where males hijack eggs to clone themselves. Phil. Trans. R. Soc. B 371, 20150534 (doi:10.1098/rstb.2015.0534) 27619698 10.1098/rstb.2015.0534 PMC5031619

[4] Schlupp I 2005 The evolutionary ecology of gynogenesis. Annu. Rev. Ecol. Evol. Syst. 36, 399–417. (doi:10.1146/annurev.ecolsys.36.102003.152629)

[5] Lehtonen J, Schmidt DJ, Heubel K, Kokko H 2013 Evolutionary and ecological implications of sexual parasitism. Trends Ecol. Evol. 28, 297–306. (doi:10.1016/j.tree.2012.12.006) 23399316 10.1016/j.tree.2012.12.006

[6] Normark BB 2009 Unusual gametic and genetic systems. In Sperm biology: an evolutionary perspective, 1st ed. (eds Birkhead TR, Hosken DJ, Pitnick S), pp. 507–545. Oxford, UK: Elsevier.

[7] Neaves WB, Baumann P 2011 Unisexual reproduction among vertebrates. Trends Genet. 27, 81–88. (doi:10.1016/j.tig.2010.12.002) 21334090 10.1016/j.tig.2010.12.002

[8] Avise JC 2015 Evolutionary perspectives on clonal reproduction in vertebrate animals. Proc. Natl Acad. Sci. USA 112, 8867–8873. (doi:10.1073/pnas.1501820112) 26195735 10.1073/pnas.1501820112 PMC4517198

[9] McKone MJ, Halpern SL 2003 The evolution of androgenesis. Am. Nat. 161, 641–656. (doi:10.1086/368291) 12776890 10.1086/368291

[10] Stanley JG 1976 Production of hybrid, androgenetic, and gynogenetic grass carp and carp. Trans. Am. Fish. Soc. 105, 10–16. (doi:10.1577/1548-8659(1976)105<10:POHAAG>2.0.CO;2)

[11] Wang Z-W, Zhu H-P, Wang D, Jiang F-F, Guo W, Zhou L, Gui J-F 2011 A novel nucleo-cytoplasmic hybrid clone formed via androgenesis in polyploid gibel carp. BMC Res. Notes 4, 82 (doi:10.1186/1756-0500-4-82) 21439093 10.1186/1756-0500-4-82 PMC3072332

[12] Morgado-Santos M, Carona S, Magalhães MF, Vicente L, Collares-Pereira MJ 2016 Reproductive dynamics shapes genomotype composition in an allopolyploid complex. Proc. R. Soc. B 283, 20153009 (doi:10.1098/rspb.2015.3009) 10.1098/rspb.2015.3009 PMC4892787 27226473

[13] Collares-Pereira MJ, Matos I, Morgado-Santos M, Coelho MM 2013 Natural pathways towards polyploidy in animals: the *Squalius alburnoides* fish complex as a model system to study genome size and genome reorganization in polyploids. Cytogenet. Genome Res. 140, 97–116. (doi:10.1159/000351729) 23796598 10.1159/000351729

[14] Lamatsch DK, Stöck M 2009 Sperm-dependent parthenogenesis and hybridogenesis in teleost fishes. In Lost sex: the evolutionary biology of parthenogenesis (eds Schön I, Martens K, van Dijk P), pp. 399–432. Berlin, Germany: Springer.

[15] Alves MJ, Gromicho M, Collares-Pereira MJ, Crespo-Lopez E, Coelho MM 2004 Simultaneous production of triploid and haploid eggs by triploid *Squalius alburnoides* (Teleostei: Cyprinidae). J. Exp. Zool. A Ecol. Genet. Physiol. 301A, 552–558. 10.1002/jez.a.51 15229865

[16] Alves MJ, Coelho MM, Collares-Pereira MJ 1998 Diversity in the reproductive modes of females of the *Rutilus alburnoides* complex (Teleostei, Cyprinidae): a way to avoid the genetic constraints of uniparentalism. Mol. Biol. Evol. 15, 1233–1242. (doi:10.1093/oxfordjournals.molbev.a025852)

[17] Alves MJ, Coelho MM, Próspero MI, Collares-Pereira MJ 1999 Production of fertile unreduced sperm by hybrid males of the *Rutilus alburnoides* complex (Teleostei, Cyprinidae): an alternative route to genome tetraploidization in unisexuals. Genetics 151, 277–283. 9872966 10.1093/genetics/151.1.277 PMC1460441

[18] Morgado-Santos M, Matos I, Vicente L, Collares-Pereira MJ 2010 Scaleprinting: individual identification based on scale patterns. J. Fish Biol. 76, 1228–1232. (doi:10.1111/j.1095-8649.2010.02591.x) 20409174 10.1111/j.1095-8649.2010.02591.x

[19] Lamatsch DK, Steinlein C, Schmid M, Schartl M 2000 Noninvasive determination of genome size and ploidy level in fishes by flow cytometry: detection of triploid *Poecilia formosa*. Cytometry 39, 91–95. (doi:10.1002/(SICI)1097-0320(20000201)39:2<91::AID-CYTO1>3.0.CO;2-4) 10679726 10.1002/(sici)1097-0320(20000201)39:2<91::aid-cyto1>3.0.co;2-4

[20] Sousa-Santos C, Robalo JI, Collares-Pereira MJ, Almada VC 2005 Heterozygous indels as useful tools in the reconstruction of DNA sequences and in the assessment of ploidy level and genomic constitution of hybrid organisms. DNA Seq. 16, 462–467. (doi:10.1080/10425170500356065) 16287626 10.1080/10425170500356065

[21] Miller SA, Dykes DD, Polesky HF 1988 A simple salting out procedure for extracting DNA from human nucleated cells. Nucleic Acids Res. 16, 1215 (doi:10.1093/nar/16.3.1215) 3344216 10.1093/nar/16.3.1215 PMC334765

[22] Sousa-Santos C, Robalo J, Almada V 2014 Spawning behaviour of a threatened Iberian cyprinid and its implications for conservation. Acta Ethol. 17, 99–106. (doi:10.1007/s10211-014-0185-5)

[23] Pires DF, Pires AM, Collares-Pereira MJ, Magalhães MF 2010 Variation in fish assemblages across dry-season pools in a Mediterranean stream: effects of pool morphology, physicochemical factors and spatial context. Ecol. Freshw. Fish 19, 74–86. (doi:10.1111/j.1600-0633.2009.00391.x)

[24] Guerrero RD, Shelton WL 1974 Aceto-carmine squash method for sexing juvenile fishes. Progress. Fish-Culturist 36, 56 (doi:10.1577/1548-8659(1974)36[56:AASMFS]2.0.CO;2)

[25] Turner TF, Dowling TE, Broughton RE, Gold JR 2004 Variable microsatellite markers amplify across divergent lineages of cyprinid fishes (subfamily Leuciscinae). Conserv. Genet. 5, 279–281. (doi:10.1023/B:COGE.0000029998.11426.ab)

[26] Muenzel FM, Sanetra M, Salzburger W, Meyer A 2007 Microsatellites from the vairone *Leuciscus souffia* (Pisces: Cyprinidae) and their application to closely related species. Mol. Ecol. Notes 7, 1048–1050. (doi:10.1111/j.1471-8286.2007.01772.x)

[27] Vyskocilova M, Simkova A, Martin J-F 2007 Isolation and characterization of microsatellites in *Leuciscus cephalus* (Cypriniformes, Cyprinidae) and cross-species amplification within the family Cyprinidae. Mol. Ecol. Notes 7, 1150–1154. (doi:10.1111/j.1471-8286.2007.01813.x)

[28] Schuelke M 2000 An economic method for the fluorescent labeling of PCR fragments. Nat. Biotechnol. 18, 233–234. (doi:10.1038/72708) 10657137 10.1038/72708

[29] Robalo JI, Almada VC, Levy A, Doadrio I 2007 Re-examination and phylogeny of the genus *Chondrostoma* based on mitochondrial and nuclear data and the definition of 5 new genera. Mol. Phylogenet. Evol. 42, 362–372. (doi:10.1016/j.ympev.2006.07.003) 16949308 10.1016/j.ympev.2006.07.003

[30] Tamura K, Stecher G, Peterson D, Filipski A, Kumar S 2013 MEGA6: molecular evolutionary genetics analysis, version 6.0. Mol. Biol. Evol. 30, 2725–2729. (doi:10.1093/molbev/mst197) 24132122 10.1093/molbev/mst197 PMC3840312

[31] Morgado-Santos M, Pereira HM, Vicente L, Collares-Pereira MJ 2015 Mate choice drives evolutionary stability in a hybrid complex. PLoS ONE 10, e0132760 (doi:10.1371/journal.pone.0132760) 26181664 10.1371/journal.pone.0132760 PMC4504517

[32] Scali V 2009 Stick insects: parthenogenesis, polyploidy and beyond. In Life and time: the evolution of life and its history (eds Casellato S, Burighel P, Minelli A), pp. 171–192. Padova, Italy: Cleup.

[33] Mason AS, Pires JC 2015 Unreduced gametes: meiotic mishap or evolutionary mechanism? Trends Genet. 31, 5–10. (doi:10.1016/j.tig.2014.09.011) 25445549 10.1016/j.tig.2014.09.011

[34] ASAB. 2015 Guidelines for the treatment of animals in behavioural research and teaching. Anim. Behav. 99, I–IX. 10.1006/anbe.1999.1349 10640387

[35] Sousa-Santos C, Collares-Pereira MJ, Almada V 2007 Reading the history of a hybrid fish complex from its molecular record. Mol. Phylogenet. Evol. 45, 981–996. (doi:10.1016/j.ympev.2007.05.011) 17600731 10.1016/j.ympev.2007.05.011

