# Supplementary material for: First empirical evidence of naturally occurring androgenesis in vertebrates
Source: R Soc Open Sci. 2017 May 24;4(5):170200. doi: 10.1098/rsos.170200 (PMC5451830; doi:10.1098/rsos.170200)
Supplement: Microsatellite details [file rsos170200supp1.doc]

**Supplementary Table 1.** Details on the ten microsatellites used for paternity analysis, including species of origin. Microsatellites marked with an asterisk (*) were amplified using primers with a M13 tail, as described in [28].

| **Microsatellite** | **Species** | **Primers (5' → 3')** | **PCR conditions** | **Reference** |
| --- | --- | --- | --- | --- |
| LCO1 | *Luxilus cornutus* | CACGGGACAATTTGGATGTTTTAT AGGGGGCAGCATACAAGAGACAAC | **MgCl_2_:** 1,00 mM  **Annealing:** 51 ºC (34 cycles) | [25] |
| LCO3 | *Luxilus cornutus* | GCAGGAGCGAAACCATAAAT AAACAGGCAGGACACAAAGG | **MgCl_2_:** 1,50 mM  **Annealing:** 48 ºC (28 cycles) | [25] |
| LCO4 | *Luxilus cornutus* | ATCAGGTCAGGGGTGTCACG TGTTTATTTGGGGTCTGTGT | **MgCl_2_:** 1,30 mM  **Annealing:** 60 ºC (31 cycles) | [25] |
| LC27* | *Leuciscus cephalus* | TCCAGTTCTTCCTTCCTAATT GCGGAGGGAGAGTATGTCAA | **MgCl_2_:** 1,00 mM  **Annealing:** 53 ºC (23 cycles), 51 ºC (10 cycles) | [27] |
| LC288* | *Leuciscus cephalus* | AAGAGCAGAGGAGAGCAGGG TACCTGCAGGGGCATAGGC | **MgCl_2_:** 1,25 mM **Annealing:** 53 ºC (23 cycles), 51 ºC (15 cycles) | [27] |
| LC290* | *Leuciscus cephalus* | CCCTAATGGCCCTCAATACA ACTTCGCTGGCTTGACAAAT | **MgCl_2_:** 1,25 mM **Annealing:** 54 ºC (25 cycles), 53 ºC (10 cycles) | [27] |
| Lsou05* | *Leuciscus souffia* | CTGAAGAAGACCCTGGTTCG CCCACATCTGCTGACTCTGAC | **MgCl_2_:** 1,25 mM **Annealing:** 55 ºC (25 cycles), 53 ºC (12 cycles) | [26] |
| Lsou08* | *Leuciscus souffia* | GCGGTGAACAGGCTTAACTC TAGGAACGAAGAGCCTGTGG | **MgCl2:** 1,25 mM **Annealing:** 55 ºC (25 cycles), 53 ºC (12 cycles) | [26] |
| Lsou34* | *Leuciscus souffia* | CCAGACAGGGTGATGATTCC GTAGCGACGTTCAGGTCTCG | **MgCl_2_:** 1,50 mM **Annealing:** 55 ºC (25 cycles), 53 ºC (8 cycles) | [26] |
| SpyrAMT* | *Squalius pyrenaicus* | GAAGAAAGTCTCATTGCTCTGC GAGGTCATCACCCACACCTT | **MgCl_2_:** 1,25 mM **Annealing:** 55 ºC (27 cycles), 53 ºC (8 cycles) | [12] |
